# Supplementary material for: Splice-Junction-Based Mapping of Alternative Isoforms in the Human Proteome
Source: Cell Rep. Author manuscript; Available in PMC 2020 Jan 15. (PMC6961840; doi:10.1016/j.celrep.2019.11.026)
Supplement: 3 [file NIHMS1546469-supplement-3.zip › DF2/PXD0010154/Colon-76-Q15063-KLLQEDTPVR.pdf]

A

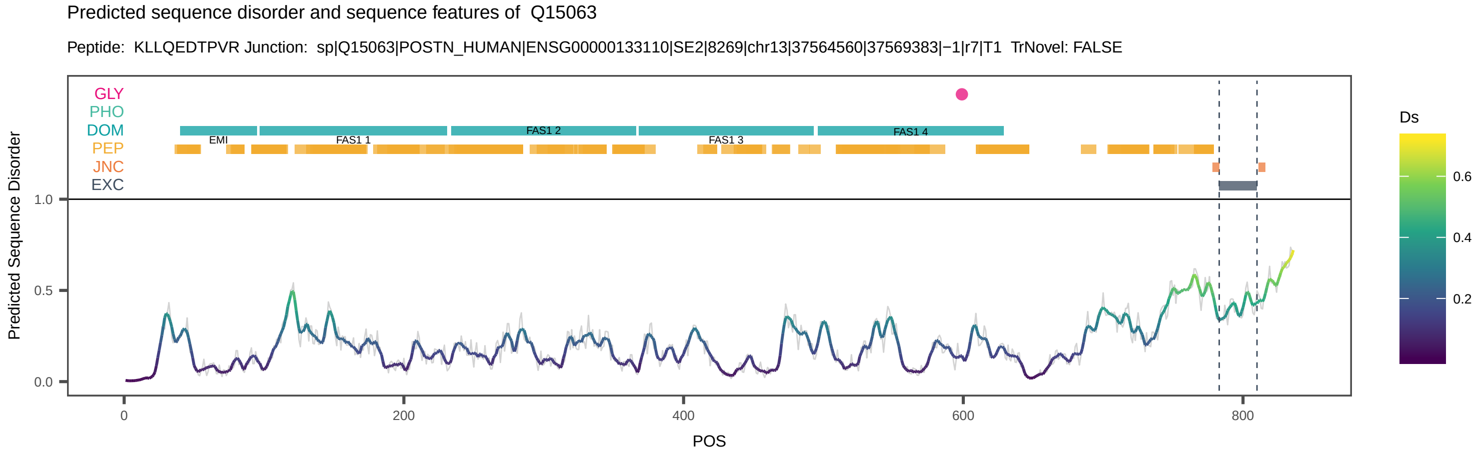
Predicted sequence disorder and sequence features of Q15063
Peptide: KLLQEDTPVR Junction: sp|Q15063|POSTN_HUMAN|ENSG00000133110|SE2|8269|chr13|37564560|37569383|−1|r7|T1 TrNovel: FALSE
GLY
PHO
DOM
PEP
JNC
EXC
EMI
FAS1 1
FAS1 2
FAS1 3
FAS1 4
Predicted Sequence Disorder
1.0
0.5
0.0
0
200
400
600
800
POS
Ds
0.6
0.4
0.2

B

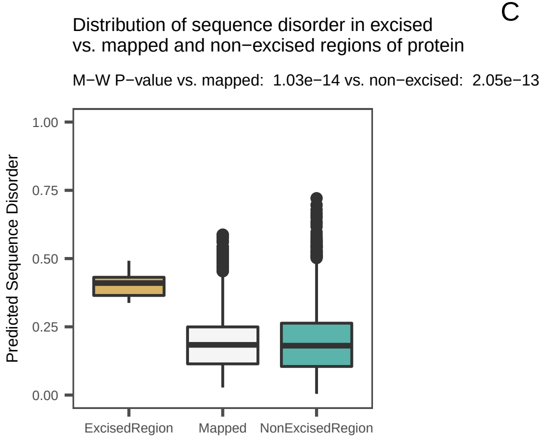
Distribution of sequence disorder in excised
vs. mapped and non−excised regions of protein
M−W P−value vs. mapped: 1.03e−14 vs. non−excised: 2.05e−13
Predicted Sequence Disorder
1.00
0.75
0.50
0.25
0.00
ExcisedRegion
Mapped
NonExcisedRegion

C
